# STM-based symbolic regression for strength prediction of RC deep beams and corbels

**DOI:** 10.1038/s41598-024-74803-9

**Published:** 2024-10-23

**Authors:** Khaled Megahed

**Affiliations:** Department of Structural Engineering, Mansoura University, PO BOX 35516, Mansoura, Egypt

**Keywords:** Machine learning, Symbolic regression, Strut-and-tie model, Deep beams, Corbels, CatBoost model, Civil engineering, Statistics, Scientific data, Computer science

## Abstract

This study uses symbolic regression with a strut-and-tie model to predict the shear strength of reinforced concrete deep beams (RCDBs) and corbels (RCCs). Previous studies have proposed two distinct types of models for estimating shear capacity: explainable models based on theoretical derivations and black-box models derived from machine learning (ML) methods. This study proposes a hybrid model derived from the strut-and-tie model (STM), where the performance of STM is enhanced through the ML approach using genetic programming. This model is based on a comprehensive experimental database of 810 tests for the shear strength of RC deep beams and 371 tests for RC corbels from various research papers. The developed STM-based symbolic regression (SR-STM) integrates two distinct force-transferring mechanisms: the diagonal strut mechanism utilizing concrete strength and the truss mechanism utilizing orthogonal web reinforcement. The SR-STM model is both robust and interpretable, demonstrating high prediction accuracy with mean values of the prediction-to-actual ratios of 0.999 and 1.004 and coefficient of determination values of 0.913 and 0.862 for RCDBs and RCCs, respectively, while providing explainable mathematical expressions that align with the mechanical principles of STM. The developed SR-STM model is benchmarked against several state-of-the-art models and evaluated against the CatBoost ML technique, demonstrating acceptable performance. The results highlight the SR-STM model’s effectiveness in providing reliable predictions and valuable insights for practical engineering applications. Furthermore, a SHAP (Shapley Additive Explanations) analysis was performed, and its results align with the SR-STM model, confirming the model’s effectiveness in accurately capturing the key factors influencing the shear strength of RCDBs and RCCs.

**Supplementary Information:**

The online version contains supplementary material available at 10.1038/s41598-024-74803-9.

## Introduction

Deep reinforced concrete (RC) elements, such as deep beams (RCDBs) and corbels (RCCs), characterized by a small span-to-height ratio^1,2^, are frequently used as load-transferring components in structures, such as transfer girders and pile caps, due to their superior shear strength compared to slender members. Despite their extensive usage, designing these elements is challenging due to the nonlinear influence of various parameters on their shear behavior. Multiple shear strength models have been studied, including those utilizing machine learning methods^3–8^, the strut-and-tie model (STM)^9–11^, the compression field method^12^, and finite element analysis^13^. Traditional design methods, such as the STM, often fail to adequately capture the complex relationships between parameters impacting shear strength, resulting in inaccurate strength prediction. Additionally, existing code provisions, such as ACI 318^14^, and EC2^15^, along with previous mechanical models^10,11^, offer straightforward procedures for calculating the shear capacity of RCDBs and RCCs. However, their conservative approach and inconsistency with test results fail in developing a comprehensive model that can accurately estimate the shear capacity of these elements.

Deep beams and corbels are frequently characterized by discontinuity regions, commonly called “D-region members.” Within these D-regions, nonlinear strain distributions arise from sudden changes in geometry or loading configurations^16^, making traditional design methods based on classical Bernoulli beam theory inadequate. The strut-and-tie model (STM) has emerged as a robust and effective approach for designing deep reinforced concrete elements. Numerous STMs have been proposed in the literature for estimating the shear capacities of the D-regions^11,17−19^. However, despite the numerous STMs proposed, some models have been criticized for their inconsistent and complex predictions of shear strength^2,4,8,19^.

Machine learning (ML) has recently become increasingly prominent in various engineering applications, offering an alternative approach to traditional mechanical theories. ML algorithms, including artificial neural networks, genetic algorithms, and ensemble learning methods, have been widely applied to predict the shear capacity of deep RC elements^3–8^. For instance, Ma et al^4^. applied six different ML models to forecast the shear capacity of RCDBs and compared their performance with previous state-of-the-art models. Feng et al^5^. investigated four standard ensemble learning models—random forests, gradient boosting regression trees, adaptive boosting, and extreme gradient boosting (XGBoost)—to estimate the shear capacity of RCDBs using a dataset comprising 271 samples. Ashour et al^3^. utilized genetic expression programming to formulate an empirical expression for the shear capacity of RCDBs relying on 141 test data points. In addition, Shahnewaz et al^6^. and Wakjira^7^employed genetic algorithm for shear strength prediction of RCDBs. However, these models are challenging to apply in practical engineering design because the purely data-driven prediction procedure cannot be translated into a feasible mathematical expression for structural application. Consequently, data-driven algorithms are often viewed as black-box models. Furthermore, many expressions derived from ML techniques, such as genetic expression programming (GEP) and genetic algorithms (GA), are often criticized for their lack of physical significance and excessive complexity^3,6,7^, as outlined in Table 1. In addition, recent studies have demonstrated the potential of ML to optimize and innovate within the field of civil engineering. Inqiad et al. used GEP for predicting the compressive strength of self-compacting concrete. Moreover, Khaled et al^20^. used different ML models to predict the axial capacity of rectangular concrete-filled concrete columns, including the Gaussian process (GPR) and the extreme gradient boosting model (XGBoost). Additionally, Moradi et al. [46] introduced a novel experimental approach to enhance the shear capacity of RC beams by utilizing fiber-reinforced polymer wraps. These advancements highlight the growing role of machine learning in improving the accuracy, efficiency, and innovation of civil engineering practices.

**Table 1:** Summary of previous GP and GA models for predicting shear strength of deep RC elements.

Reference	Number	Models: Statistical criteria
Wakjira 2020^7^	371	**GP**:$\:{V}_{th}=0.0456{{f}_{c}^{{\prime\:}}}^{0.619}{\rho\:}_{l}^{0.411}{\left(\frac{a}{d}\right)}^{-0.874}{b}_{w}d$µ = 0.82, COV = 0.305 (for RCDBs without web reinforcement)
Ashour 2003^3^	141	**GP**:$\:V={b}_{w}h\sqrt{{f}_{c}^{{\prime\:}}}\left[\left(-4.56+1.68\frac{a}{d}\right){\rho\:}_{l}^{2}+\left(2.45+0.1{\left(\frac{a}{d}\right)}^{2}-1.16\frac{a}{d}+3.12{\rho\:}_{t}\right){\rho\:}_{l}+0.3{\rho\:}_{hw}+0.4{\rho\:}_{vw}\right]$µ = 1.11, Std =0.21 (for RCDBs)
Shahnewaz 2020^6^	381	**GA**:$\:{V}_{u}={b}_{w}h{f}_{c}^{{\prime\:}}\left[\frac{2}{5}-\frac{1}{4}{\left(\frac{a}{d}\right)}^{0.23}+0.85{\left({\rho\:}_{l}{\rho\:}_{hw}{\rho\:}_{vw}\right)}^{0.1}-\frac{3}{5}{\left(\left(\frac{a}{d}\right){\rho\:}_{hw}{\rho\:}_{vw}\right)}^{\frac{1}{16}}-200{\left(\left(\frac{a}{d}\right){\rho\:}_{l}{\rho\:}_{hw}{\rho\:}_{vw}\right)}^{2.65}\right]$µ = 0.99, CoV = 0.232 (for RCDBs) **GA**:$\:{V}_{u}={b}_{w}h{f}_{c}^{{\prime\:}}\left[1.74-2{\left(\frac{a}{d}\right)}^{0.044}+0.5{\rho\:}^{0.14}\right]$µ = 1.01, CoV = 0.257 (for RCDBs)

Due to the differing prediction mechanisms of explainable models (e.g., strut-and-tie models) and black-box models (e.g., data-driven models), these approaches have traditionally been considered independent in resistance prediction^3–8^. Previous studies have favored strut-and-tie models for their interpretable mechanisms, while black-box models have been preferred for their superior performance. This study uses a machine-learning-based symbolic regression (SR) technique to develop an ML-aided STM for predicting the shear strength of RCDBs and RCCs. This technique serves as a hybrid model or an intermediate solution, effectively bridging the gap between mechanical-based and black-box models, thereby gaining popularity in recent studies^21,22^. Given the robust performance of STM in estimating the shear capacity of deep RC elements, integrating the SR technique can calibrate the STM, resulting in a hybrid model with high prediction accuracy.

This study aims to develop a symbolic regression model based on the Strut-and-Tie Model (SR-STM) to predict the shear strength of RCDBs and RCCs precisely. The novelty of this research lies in integrating the symbolic regression (SR) technique with the robust performance of STM. An enforced structure tree for symbolic regression is utilized to achieve three primary objectives: (1) reduce the search space, thereby enhancing the efficiency of the genetic programming process; (2) provide explainable expressions consistent with the Strut-and-Tie Model; and (3) diverge from previous research that generated uninterpretable functions using SR. The term “interpretability” in this context refers to the model’s ability to produce equations that are not only computationally feasible but also aligned with the physical behavior of the structures, making them meaningful and actionable for engineers. This approach ensures that the resulting models are not only computationally feasible but also maintain interpretability and consistency with established mechanical principles. Additionally, the developed model was calibrated against a comprehensive database of test results, including 810 deep beams and 371 corbels, which is more extensive than those considered by state-of-the-art models^11^^,^^17^^–^^19^. The full list of input features, including geometric configurations, concrete properties, reinforcement characteristics, and loading plate dimensions, is detailed in the next section (see the Experimental Database section). Various metrics were applied to evaluate the model’s accuracy and generality. Finally, the predictions obtained from the proposed STM-based model were compared with those from the CatBoost ensemble machine learning technique, known for its superior performance^8^, and three existing closed-form models^11^^,^^17^^–^^19^. This comparison highlights the effectiveness and reliability of the proposed model in predicting shear strength while ensuring interpretability and alignment with mechanical principles.

## Experimental database

**Figure 1:**
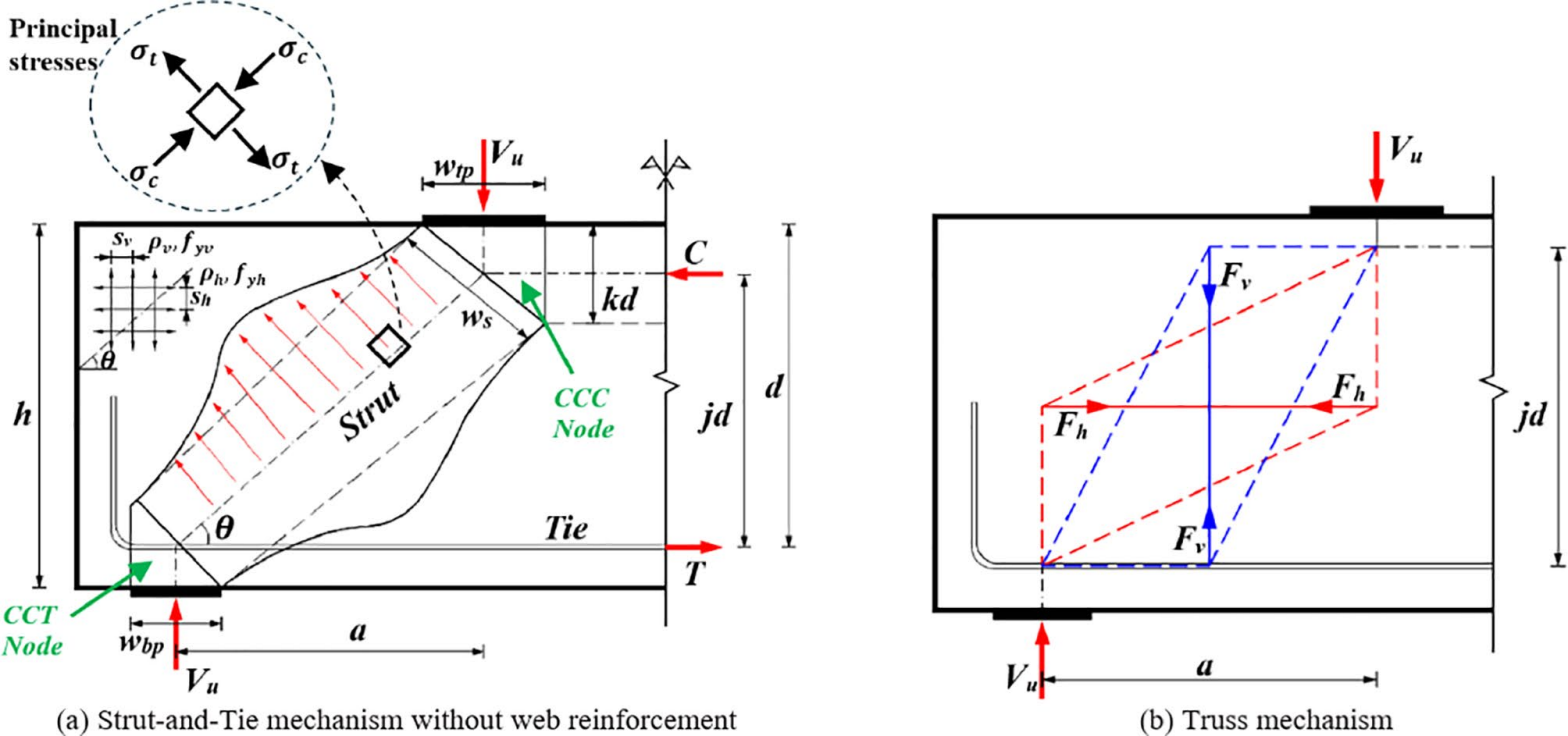
The dimensions of RC deep beam and force transfer-mechanisms.

**Figure 2:**
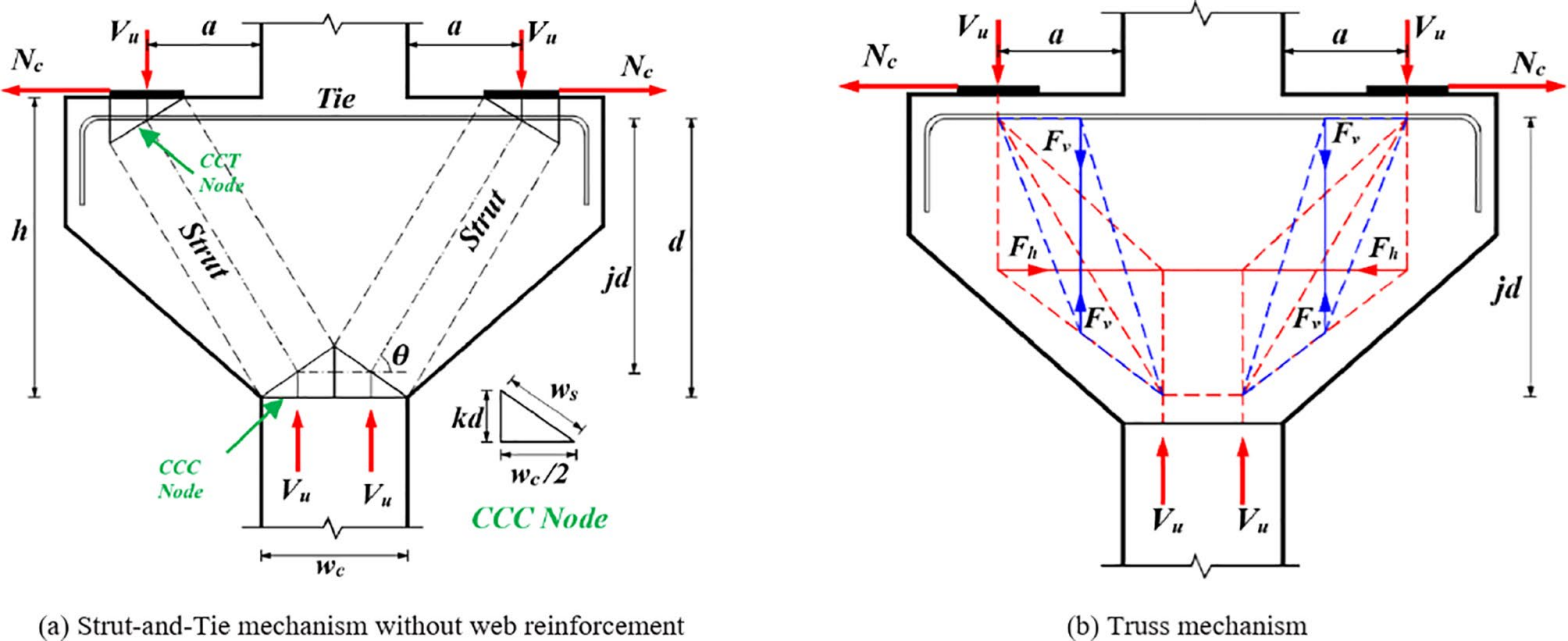
The dimensions of RC doubly symmetric corbel and force transfer-mechanisms.

The schematic diagrams of the shear mechanisms of RCDBs and RCCs are illustrated in Figs. 1 and 2, respectively. A comprehensive dataset was collected to develop ML models, consisting of 810 RCDB and 371 RCC experiments sourced from existing literature and a database gathered by Chetchotisak et al^19^. Detailed information about the collected database is available in the supplementary data. Numerous experimental and theoretical studies^9,11,17,18^, have identified that the shear capacity of deep RC elements is influenced by various factors, categorized into six groups: (1) geometric configurations: beam height (*h*), effective height (*d*), width (*b*_*w*_), shear span (*a*) and shear span-to-depth ratio (*a/d*); (2) concrete properties, i.e., concrete strength (*f*_*c*_*’*); (3) bottom longitudinal reinforcement characteristics: reinforcement ratio (*ρ*_*l*_), and strength (*f*_*yl*_); (4) web reinforcement characteristics: vertical web reinforcement ratio (*ρ*_*v*_) and strength (*f*_*yv*_), horizontal web reinforcement ratio (*ρ*_*h*_) and strength (*f*_*yh*_); (5) loading plate dimensions: top plate width (*w*_*tp*_) and bottom plate width (*w*_*bp*_); (6) horizontal force for corbels (*N*_*c*_). Table 2 summarizes statistical information for the established database.

**Table 2:** Statistic features of the experimental dataset.

Variable	Symbol	Type	Statistics (RCDBs)				Statistics (RCCs)			
			Min	Max	Mean	Std	Min	Max	Mean	Std
Beam height	$\:h$ (mm)	Input	160	2100	552	279	120	1143	395	185
Beam effective height	$\:d$ (mm)	Input	140	2000	488	260	92	1059	352	171
Beam width	$\:{b}_{w}$ (mm)	Input	51	914	194	120	115	600	184	57
Shear span	$\:a$ (mm)	Input	80	4375	618	445	50	870	184	128
Shear span-to-depth ratio	$\:a/d$	Input	0.27	2.5	1.29	0.54	0.11	1.69	0.59	0.35
Concrete strength	$\:{f}_{c}^{{\prime\:}}$ (MPa)	Input	11.3	120.1	40.4	21.5	15	105	43	18.4
Bottom reinforcement ratio	$\:{\rho\:}_{l}$ (%)	Input	0.26	11.33	2	1.11	0.21	4.93	1.24	0.76
Bottom reinforcement strength	$\:{f}_{yl}$ (MPa)	Input	267	1330	468	130	298	1480	433	131
Vertical web reinforcement ratio	$\:{\rho\:}_{v}$ (%)	Input	0	3.17	0.17	0.35	0	2.33	0.4	0.44
Vertical web reinforcement strength	$\:{f}_{yv}$ (MPa)	Input	0	855	211	230	0	760	270	235
Horizontal web reinforcement ratio	$\:{\rho\:}_{h}$ (%)	Input	0	2.86	0.29	0.37	0	1.24	0.09	0.26
Horizontal web reinforcement strength	$\:{f}_{yh}$ (MPa)	Input	0	1051	280	226	0	750	78	197
Shear strength index	$\:{v}_{n}=\frac{{V}_{u}}{{b}_{w}h{f}_{c}^{{\prime\:}}}$	Output	0.03	0.29	0.14	0.05	0.05	0.34	0.16	0.05

### Strut-and-tie model

Figures 1 and 2 illustrate two load-transferring mechanisms in a typical D-region for RCDB and RCC members: the diagonal strut mechanisms (Figs. 1(a), 2(a)) and truss mechanisms (Figs. 1(b), 2(b)). In the diagonal strut mechanisms, the primary force-transferring system consists of diagonal compression struts (depicted as dashed lines) and tension ties (depicted as solid lines), which intersect at nodes called nodal zones. While the truss mechanism describes how the shear force is resisted by the orthogonal shear reinforcement^11,23^. This mechanism is composed of two subtrusses: the horizontal subtruss, where tension forces are transferred by the horizontal web reinforcement (red lines), and the vertical subtruss, where tension forces are transferred by the vertical web reinforcement (blue lines). The term *θ* in Figs. 1 and 2 represent the angle of the diagonal concrete strut relative to the horizontal plane and can be calculated using Eq. (1).

1 $$\:\text{tan}\;\theta\:=\frac{jd}{a}$$

where $\:jd=d-kd/3$is the height of the moment arm^11,17^, with *d* being the effective depth and *kd*​ the depth of the compression zone of the member. The term *kd*can be derived from the elastic bending theory^11,17^ as:

2 $$\:kd=\left(\sqrt{{\left(n{\rho\:}_{l}\right)}^{2}+2\left(n{\rho\:}_{l}\right)}-\left(n{\rho\:}_{l}\right)\right)d$$

where *n* represents the ratio of Young’s modulus of steel to concrete. For the case of RCC under a horizontal force *N*_*c*_ (Fig. 2), the term *ρ*_*l*_ in Eq. (2) is assumed by previous studies^17,19,23^ as:

3 $$\:{\rho\:}_{l}=\frac{{A}_{l}-{N}_{c}/{f}_{yl}}{{b}_{w}d}$$

where *A*_*l*_ is the cross-sectional area of the longitudinal steel reinforcement.

Generally, an STM consisting of diagonal strut and truss mechanisms is considered to be a statically indeterminate structure^11,17,18,23^. For simplicity, the shear strength of deep RC element (*V*_*n*_) is assumed to be the sum of the shear strengths provided by the diagonal concrete strut (*V*_*c*_) and truss mechanisms (*V*_*w*_), as established by previous studies^10,11,17^, as follows:

4 $$\:{V}_{n}={V}_{c}+{V}_{w}$$

## Shear strength contributed by diagonal strut mechanism

According to the STM approach, the brittle shear failure in deep RC elements is primarily governed by the biaxial strength of the diagonal struts, which is usually less than the strength of the nodal regions^18^. Many studies^13^^,^^24^^−^^26^ have employed the Mohr–Coulomb failure criterion for defining the biaxial strength of diagonal struts, as follows:

5 $$\:\frac{{\sigma}_{t}}{{f}_{t}}+\frac{{\sigma}_{c}}{{f}_{c}^{{\prime\:}}}=1.0$$

where *f*_*t*_ is the concrete tensile strength and *σ*_*t*_ and *σ*_*c*_ indicate the principal tensile and compressive stresses inside the diagonal strut, respectively, as shown in Fig. 1(a). For simplicity, the stress ratio *σ*_*t*_/*f*_*t*_ in Eq. (5) is assumed as the ratio of the tie tension force *T*to the tensile strength of longitudinal steel bars^13,19^ as follows:

6 $$\:\frac{{\sigma\:}_{t}}{{f}_{t}}\approx\:\frac{T}{\left({\rho\:}_{l}{b}_{w}d\right){f}_{yl}}$$

As depicted in Fig. 1(a), the tension force T at the CCT node (node surrounded by two struts and one tie) is calculated through the balance of horizontal forces as follows:

7 $$\:T={V}_{c}/\text{tan}\;\theta\:$$

While the diagonal compressive stress $\:{\sigma\:}_{c}$ acting on the concrete strut is given by:

8 $$\:{\sigma}_{c}=\frac{{C}_{c}}{{A}_{str}}=\frac{{V}_{c}}{{A}_{str}\text{sin}\;\theta\:}$$

where *A*_*str*_ is diagonal strut area as illustrated in Figs. 1(a) and 2(a) by the following expression:

9 $$\:{A}_{str}={b}_{w}\sqrt{{\left(kd\right)}^{2}+{\left({w}_{l}\right)}^{2}}$$

where *w*_*l*_is the projected horizontal width of the CCC node (node surrounded by three struts)^11,17,19,23,27^. For simplicity in this study, *w*_*l*_ is assumed to be equal to the width of the top plate for deep beams and *w*_*c*_/2 for doubly symmetric corbels, where *w*_*c*_ is the column width (Fig. 2(a)).

By substituting Eqs. (6) and (8) into Eq. (5), the shear capacity​ of the diagonal strut mechanism (*V*_*c*_) can be expressed as:

10 $$\:\frac{1}{{V}_{c}}=\frac{1}{{V}_{s}}+\frac{1}{{V}_{t}}$$

where *V*_*s*_ and *V*_*t*_ define the strength of the diagonal strut and tension tie, respectively, given by:

11 $$\:{V}_{s}={f}_{c}^{{\prime\:}}{A}_{str}\text{sin}\;\theta\:,\:{V}_{t}={\rho\:}_{l}{b}_{w}d{f}_{yl}\text{tan}\;\theta\:$$

Equation (10) expresses the strength contributions of the concrete strut *V*_*s*_ and the tension tie *V*_*t*_ to the overall shear capacity. Rewriting Eq. (10) in a dimensionless form results in:

12 $$\begin{aligned}\frac{1}{{v}_{c}}=\frac{1}{{k}_{s}\text{sin}\;\theta\:}+\frac{1}{{\rho}_{l}^{e}\text{tan}\;\theta\:}\end{aligned}$$

where

13 $$\:{v}_{c}=\frac{{V}_{c}}{{b}_{w}d{f}_{c}^{{\prime\:}}},\:\:{k}_{s}=\sqrt{{\left(k\right)}^{2}+{\left({w}_{l}/d\right)}^{2}},\:\:{\rho}_{l}^{e}={\rho}_{l}\left(\frac{{f}_{yl}}{{f}_{c}^{{\prime\:}}}\right)$$

The term *k*_*s*_ defines the ratio of strut width to beam depth.

## Shear strength contributed by truss mechanism

According to the force equilibrium in the truss mechanism^18,27^ in Figs. 1(b) and  2(b), the shear strength *V*_*w*_ can be written as:

14 $$\:{V}_{w}={F}_{h}\text{tan}\;\theta\:+{F}_{v}$$

where *F*_*h*_ and *F*_*v*_ refer to the average tensile forces carried by the horizontal and vertical web reinforcements, respectively, in the D-regions. Accordingly, in a manner similar to Eq. (12), the shear strength of the truss mechanism *V*_*w*_ can be rewritten in dimensionless form as follows:

15 $$\:{v}_{w}=\frac{{V}_{w}}{{b}_{w}d{f}_{c}^{{\prime\:}}}={\rho}_{h}^{e}\text{tan}\:\theta\:+{\rho}_{v}^{e},\:\:with\:\:\:{\rho}_{h}^{e}={\rho}_{h}\left(\frac{{f}_{yh}}{{f}_{c}^{{\prime\:}}}\right),\:\:{\rho}_{v}^{e}={\rho}_{v}\left(\frac{{f}_{yv}}{{f}_{c}^{{\prime\:}}}\right)$$

From Eqs. (12) and (15), the overall contribution of the diagonal strut and truss mechanisms can be formulated as:

16 $$\:{V}_{n}=\left(\frac{1}{\frac{1}{{k}_{s}\text{sin}\;\theta\:}+\frac{1}{{\rho}_{l}^{e}\text{tan}\;\theta\:}}+{\rho}_{h}^{e}\text{tan}\;\theta\:+{\rho}_{v}^{e}\right){b}_{w}d{f}_{c}^{{\prime\:}}$$

## Symbolic regression

Symbolic regression (SR)^28,29^is a genetic programming technique^30^designed to discover simple and interpretable analytic equations that best fit a given problem through exploring a predefined space of mathematical expressions and functions. SR is approached as a multi-objective optimization problem, balancing predictive accuracy and model complexity. Genetic programming techniques, including the principles of natural selection and evolution, are commonly used in SR to iteratively refine candidate mathematical expressions until satisfactory expressions are achieved. This research employs a Python library called PySR^31^ to identify interpretable, simple expressions for the shear strength of RCDBs and RCCs.

**Figure 3:**
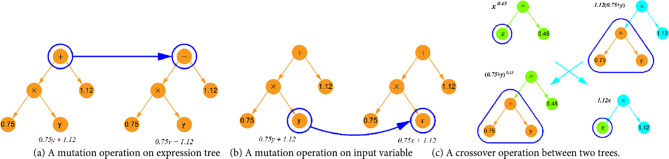
Mutation and crossover operations in SR model.

**Figure 4:**
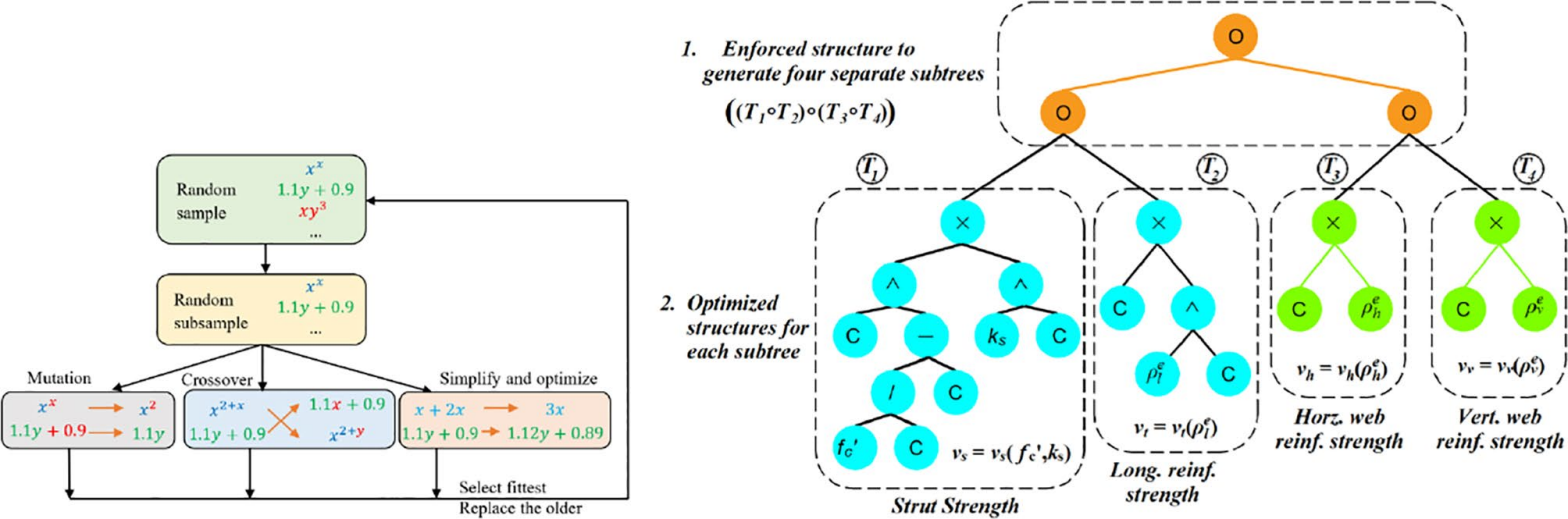
(a) Flow charts of Symbolic regression. (b) The optimal tree-based individuals for RC deep beam strength, where operator O, in the forced structure part, can be any arbitrary binary operator while, in the optimized structure part, variable C is constant.

The SR algorithm starts with creating an initial population composed of a random combination of operational symbols (e.g., +, -, *,/, ^, etc.) and terminals, such as input variables and constants. Each individual in the population is represented by a tree-like expression. Selection is then performed probabilistically, favoring those individuals that demonstrate superior performance. To prevent the generation of overly complex individuals by SR, a complexity limit of 30 is set, meaning the total number of operators, constants, and variables in the equations cannot exceed this value. In addition, overly complex expressions are excluded from the SR expressions such as high exponential terms, e.g. (•)^(•^•). The selected individuals undergo mutation (Fig. 3(a, b)) or crossover (Fig. 3(c)) to produce a new generation of populations. Figure 4(a) presents the core steps of the SR approach. This evolutionary process employs a fitness function, defined in Eq. (17)^31^, to evaluate and identify the most optimal individuals in each generation, ensuring the progressive refinement of solutions.

17 $$\:l\left(E\right)={l}_{pred}\left(E\right).\text{exp}\left(\text{f}\text{r}\text{e}\text{c}\text{e}\text{n}\text{c}\text{y}\left[C\left(E\right)\right]\right)$$

where *l*_*pred*_(*E*) represents the model prediction error, *C*(*E*) denotes the expression complexity *E*, quantified by the total number of nodes in the expression. The term frecency [*C*(*E*)] accounts for the frequency and recency of the expression *E* occurring at complexity *C*(*E*) within the population. This measure is crucial for avoiding the overcomplication and redundancy of the generated expressions, ensuring a balance between minimizing error and maintaining simplicity. Details of the SR parameters used for generating expressions in this study are summarized in Table 3.

**Table 3:** The parameters of the SR model used in generating expressions.

Parameters	Value	Parameters	Value
Number of generations	200	Allowed Binary operators	-, +, *, ^, /
Total number of populations	20	Loss function	Algorithm 1
Population size	50	Constraints	{‘^’:(–1,10)}^(a)^
Maximum length of expressions (total number of nodes)	30	Nested constraints	‘^’:{‘^’:0,’/’:1}^(b)^
Parsimony (factor controls the expression complexity)	0.02	model_selection	Accuracy

^(a)^The ‘^’:(–1,10) constraint means that the left argument of the power function can exhibit any level of complexity, whereas the right argument is restricted to a maximum complexity of 10 nodes.

^(b)^Nested constraints govern how operators can be combined or nested. The constraint ‘^’:{‘^’:0,’/’:1} specifies that ‘^’ operator cannot be used inside another ‘^’ operator, but ‘/’ operator can be nested once in ‘^’ operator.

The process of finding the best expression requires many iterations and a detailed assessment of each one. Each equation generated through these iterations is subjected to thorough evaluation and refinement, considering factors such as the complexity of the equations, their accuracy, and their ease of interpretation.

The process of finding the best expression requires many iterations, where different parameters, such as the number of generations, total population, and population size, are varied to generate distinct equations. Each equation is then subjected to a detailed assessment and refinement, considering complexity, accuracy, and ease of interpretation factors. This iterative approach ensures that the final model balances interpretability with predictive accuracy.

## STM-based symbolic regression development

The primary objective of this section is to utilize symbolic regression (SR) to refine and calibrate the strut-and-tie model (SR-STM) for predicting the shear strength of reinforced concrete deep beams (RCDBs) and corbels (RCCs). Unlike purely data-driven approaches where machine learning models determine prediction outcomes directly from data, the SR-based calibration method presented here fundamentally integrates the mechanical principles of STM with machine learning techniques to enhance prediction accuracy. This hybrid approach merges the physical insights of STM with the optimization capabilities of symbolic regression to achieve precise and meaningful prediction outcomes.

Numerous expressions for predicting the shear strength of D-regions have been developed based on STM concepts, varying in complexity and accuracy, as summarized in Table 4. For instance, Russo et al^11,17^. proposed a nonlinear contribution factor, $\:\chi\:\left({f}_{c}^{{\prime\:}}\right){f}_{c}^{{\prime\:}}$, to account for concrete strength in the strut capacity. Similarly, Hwang and Lee^18^, and Chetchotisak et al^19^. developed nonlinear expressions for strut capacity. For the remaining elements, such as the tie and web reinforcement contributions, they assumed linear partial contributions with coefficients less than 1.0 for each component. This partial contribution arises from the fact that the stresses in the web reinforcements may not reach their yield strengths simultaneously.

**Table 4:** Summary of previous mechanical models in predicting shear strength.

Previous models	Formulas
Hwang and Lee^18^	$$\:{V}_{Hwang\:}=K\xi\:{A}_{str}\text{sin}\;\theta\:,\:$$$$\:\xi\:=3.35/\sqrt{{f}_{c}^{{\prime\:}}}\le\:0.52$$$$\quad K$$ is factor accounting for web reinforcement
Russo et al^11,17^.	$\:{V}_{Russo}={k}_{c}\left(k\chi\:{f}_{c}^{{\prime\:}}\text{sin}\;\theta\:+{k}_{h}{\rho\:}_{h}{f}_{yh}\text{tan}\;\theta\:+{k}_{v}a/d{\rho\:}_{v}{f}_{yv}\right){b}_{w}d,\:\:\:\:\chi\:=0.74{r}^{3}-1.28{r}^{2}+0.22r+0.87,\:r={f}_{c}^{{\prime\:}}/105$ For deep beams, $\:{k}_{c}=0.76,{k}_{h}=0.25,\:{k}_{v}=0.35$ For corbels, $\:{k}_{c}=0.8,\:\:\:{k}_{h}=0.65,\:{k}_{v}=0$
Chetchotisak et al. (MIST)^19^	$\:{V}_{MIST}={V}_{c}+{F}_{h}{tan}\;\theta\:+{F}_{v}$ $\:{V}_{c}=\frac{1}{\frac{1}{{V}_{str}}+\frac{1}{{V}_{tie}}}\le\:0.85{f}_{c}^{{\prime\:}}{A}_{str}\text{sin}\:\theta\:,\:{F}_{h}={k}_{h}{A}_{h}{f}_{yh},\:{F}_{v}={k}_{v}{A}_{v}{f}_{yv},$ $\:{V}_{str}=\lambda\:{\left({f}_{c}^{{\prime\:}}\right)}^{\beta\:}{A}_{str}\text{sin}\theta\:,\:{V}_{tie}=\alpha\:\left({A}_{l}{f}_{yt}+0.5\sqrt{{f}_{c}^{{\prime\:}}}{w}_{t}{b}_{w}\right)\text{tan}\theta\:$ For deep beams, $\:\lambda\:=1.864,\:\beta\:=0.704,\alpha\:=3.512,\:{k}_{h}=0.161,\:{k}_{v}=0.153$ For corbels, $\:\lambda\:=1.039,\:\beta\:=0.847,\:\alpha\:=2.973,{k}_{h}=0.389,\:{k}_{v}=0.215$ $\:\text{w}\text{i}\text{t}\text{h}\:{A}_{str}={b}_{w}\sqrt{{\left(kd\right)}^{2}+{\left({w}_{l}\right)}^{2}}\:,{w}_{t}=2\left(h-d\right)$
Current study	$\:{V}_{n}=\left(\frac{1}{\frac{1}{{v}_{s}\text{sin}\;\theta\:}+\frac{1}{{v}_{t}\text{tan}\;\theta\:}}+{v}_{h}\text{tan}\;\theta\:+{v}_{v}\right){b}_{w}d{f}_{c}^{{\prime\:}}$ For deep beams, $\:{v}_{s}={0.8}^{{\alpha}_{d}}{k}_{s}^{1.1},\:\:\:\:\:\:{v}_{t}=1.6{\left({\rho}_{l}^{e}\right)}^{0.7},\:\:\:\:\:{v}_{h}=0.14{\rho}_{h}^{e},\:\:\:\:\:\:\:\:\:\:\:\:{v}_{v}=0.31{\rho}_{v}^{e}$ For corbels, $\:{v}_{s}={0.8}^{{\alpha\:}_{c}}{k}_{s}^{0.78},\:\:\:\:\:{v}_{t}=3.0{\rho\:}_{l}^{e},\:\:\:\:\:\:\:\:\:\:\:\:\:{v}_{h}=0.17{\left({\rho\:}_{h}^{e}\right)}^{0.83},\:\:{v}_{v}=0.27{\rho\:}_{v}^{e}$ $\:\text{w}\text{i}\text{t}\text{h}\:{\alpha\:}_{d}={f}_{c}^{{\prime\:}}/30-0.15,\:{\alpha\:}_{c}={f}_{c}^{{\prime\:}}/60+3.1\:,{k}_{s}=\sqrt{{k}^{2}+{\left({w}_{l}/d\right)}^{2}},{\rho\:}_{l}^{e}={\rho\:}_{l}\left(\frac{{f}_{l}}{{f}_{c}^{{\prime\:}}}\right),{\rho\:}_{h}^{e}={\rho\:}_{h}\left(\frac{{f}_{h}}{{f}_{c}^{{\prime\:}}}\right),\:{\rho\:}_{v}^{e}={\rho\:}_{v}\left(\frac{{f}_{v}}{{f}_{c}^{{\prime\:}}}\right)$

where *K* is strut-and-tie index accounting for the influence of the web reinforcement., *w*_*t*_ is the widths the tie.

In this study, symbolic regression is employed to optimize the coefficient expressions for various components of the STM: the strut (*k*_*s*_), tie ($\:{\rho}_{l}^{e}$), vertical web reinforcement ($\:{\rho}_{v}^{e}$), and horizontal web reinforcement ($\:{\rho}_{h}^{e}$) as defined in Eq. (16). The expression employed for the optimization of the STM-based symbolic regression process is as follows:

18 $$\:{V}_{n}=\left(\frac{1}{\frac{1}{{v}_{s}\left({f}_{c}^{{\prime\:}},\:{k}_{s}\right)\text{sin}\;\theta\:}+\frac{1}{{v}_{t}\left({\rho}_{l}^{e}\right)\text{tan}\;\theta\:}}+{v}_{h}\left({\rho}_{h}^{e}\right)\text{tan}\;\theta\:+{v}_{v}\left({\rho}_{v}^{e}\right)\right){b}_{w}d{f}_{c}^{{\prime\:}}$$

where the coefficient expressions for each component of the STM that need to be optimized by SR technique are:

19 $$\:{v}_{s}={v}_{s}\left({f}_{c}^{{\prime\:}},\:{k}_{s}\right),\:\:{v}_{t}={v}_{t}\left({\rho}_{l}^{e}\right),\:\:{v}_{h}={v}_{h}\left({\rho}_{h}^{e}\right),\:\:{v}_{v}={v}_{h}\left({\rho}_{v}^{e}\right)$$

with: $$\:{k}_{s}=\sqrt{{k}^{2}+{\left({w}_{l}/d\right)}^{2}},{\rho}_{l}^{e}={\rho}_{l}\left(\frac{{f}_{yl}}{{f}_{c}^{{\prime\:}}}\right),{\rho}_{h}^{e}={\rho}_{h}\left(\frac{{f}_{yh}}{{f}_{c}^{{\prime\:}}}\right),\:{\rho}_{v}^{e}={\rho}_{v}\left(\frac{{f}_{yv}}{{f}_{c}^{{\prime\:}}}\right)$$  

Symbolic regression is employed to optimize the four expressions in Eq. (19) using the PySR library. This process involves developing a custom objective loss function designed explicitly for symbolic regression, which predefines the form of the shear strength equation with the four expressions. The symbolic trees accepted in this process must have of a structure of $\:\left(\left({T}_{1}\circ\:{T}_{2}\right)\circ\:\left({T}_{3}\circ\:{T}_{4}\right)\right)$, where subtrees *T*_*1*_, *T*_*2*_, *T*_*3*_, and *T*_*4*_ define the four functions *v*_*s*_, *v*_*t*_, *v*_*h*_, and *v*_*v*_, respectively, and operator ∘ could be any arbitrary binary operator. This structure is referred to as the enforced structure of the tree generated using the developed objective function, as illustrated in Fig. 4(b). The details of the objective function are illustrated in Algorithm 1. The objective function enforces constraints on the structure of the symbolic expressions and penalizes undesirable characteristics.

As explained in Algorithm 1, the degree of the tree head is checked, and if it is not equal to two, meaning it should combine two subtrees, TL = $\:\left({T}_{1}\circ\:{T}_{2}\right)$ and TR = $\:\left({T}_{3}\circ\:{T}_{4}\right)$, a significant penalty of 1000 is imposed. The left subtree (TL) and right subtree (TR) are also checked for a degree of 2, with a smaller penalty of 100 imposed if they do not meet this criterion. The left child of the left subtree (TLL or *T*_*1*_) represents *v*_*s*_ (strut contribution) and must be a function of *f*_*c*_*’* (concrete strength) and the ratio *k*_*s*_. If *v*_*s*_ contains invalid features or negative values, penalties are added in proportion to the number of violations. Similarly, the right child of the left subtree (TLR or *T*_*2*_) is processed as *v*_*t*_ (tie contribution), which should only be a function of $\:{\rho\:}_{l}^{e}$. The contributions from the left subtree (TLL, TLR) are combined to derive the concrete contribution. Similarly, the right subtree (TR) should be branched into two children (TRL, TRR) or (*T*_*3*_, *T*_*4*_), represent the contributions of horizontal and vertical web reinforcement, respectively. It should be noted that the penalty term increases progressively by how far it deviates from the constraints, effectively guiding the genetic algorithm towards the correct factorization. Finally, the loss function combines the penalty terms and the Mean Absolute Percentage Error (MAPE) of the predictions.

The optimal tree-based individuals (Fig. 4(b)) fitting the training experimental database for RC deep beams is

20 $$\:{v}_{s}={0.8}^{{\alpha\:}_{d}}{k}_{s}^{1.1},\:\:\:\:\:\:{v}_{t}=1.6{\left({\rho}_{l}^{e}\right)}^{0.7},\:\:\:\:\:{v}_{h}=0.14{\rho}_{h}^{e},\:\:\:\:\:\:\:\:\:\:\:\:{v}_{v}=0.31{\rho}_{v}^{e}$$

where $$\:{\alpha\:}_{d}={f}_{c}^{{\prime\:}}/30-0.15$$.

The resulting training for RC corbel beams is

21 $$\:{v}_{s}={0.8}^{{\alpha}_{c}}{k}_{s}^{0.78},\:\:\:\:\:{v}_{t}=3.0{\rho}_{l}^{e},\:\:\:\:\:\:\:\:\:\:\:\:\:{v}_{h}=0.17{\left({\rho}_{h}^{e}\right)}^{0.83},\:\:{v}_{v}=0.27{\rho}_{v}^{e}$$

where $$\:{\alpha\:}_{c}={f}_{c}^{{\prime\:}}/60+3.1$$.

The optimized coefficients (e.g., *v*_*s*_, *v*_*t*_, *v*_*h*_, *v*_*t*_) reflect the interplay between these factors and the overall shear strength. For instance, the strut contribution coefficient expression (*v*_*s*_) is proportional to the ratio of strut width to beam depth (*k*_*s*_). In addition, *v*_*s*_is inversely proportional to the concrete compressive strength due to the brittle behavior of high-strength concrete. This result agrees with the expression provided by Russo et al^11,17^. in Table 4, as the variable *χ* is inversely proportional to the concrete compressive strength. The remaining coefficients are proportional to their influencing variable. The tie contribution coefficient *v*_*t*_ is proportional to tie reinforcement ($\:{\rho\:}_{l}^{e}$), contribution coefficient *v*_*v*_ is proportional to vertical web reinforcement ($\:{\rho\:}_{v}^{e}$), and contribution coefficient *v*_*h*_ is proportional to horizontal web reinforcement ($\:{\rho\:}_{h}^{e}$), which aligns with the expected physical behavior in STM-based design. In addition, the positive impact of the diagonal strut angle *θ*aligns with experimental findings by Kani^32^, which show that beams exhibit higher shear resistance at higher angles *θ* (lower *a/d* ratios). Design examples illustrating the above equations can be found in the supplementary data.

The developed expressions for the SR-STM model are not only simple and robust but also carry physical significance, in contrast to the GEP and GA models from previous studies (see Table 1). Moreover, the enforced structure tree for symbolic regression reduces the search space and ensures that the resulting expressions are both explainable and consistent with the Strut-and-Tie Model (STM).

### Performance and results of the SR-STM model

**Figure 5:**
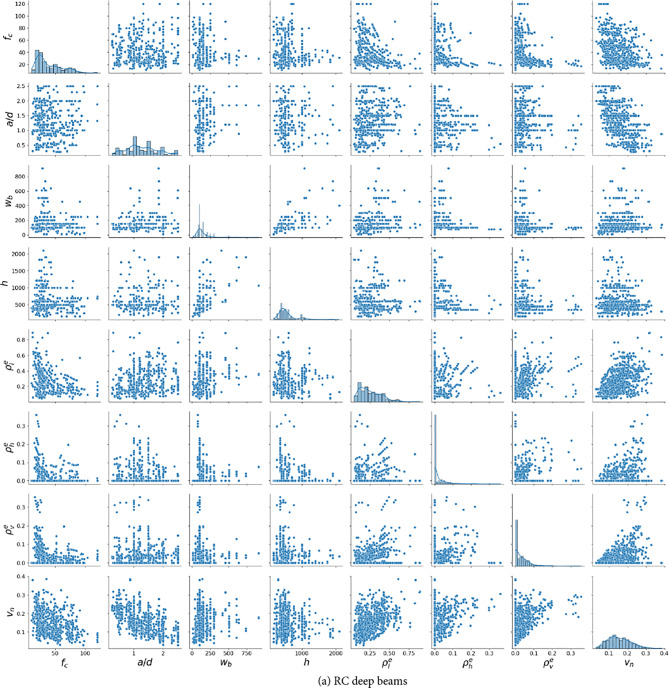

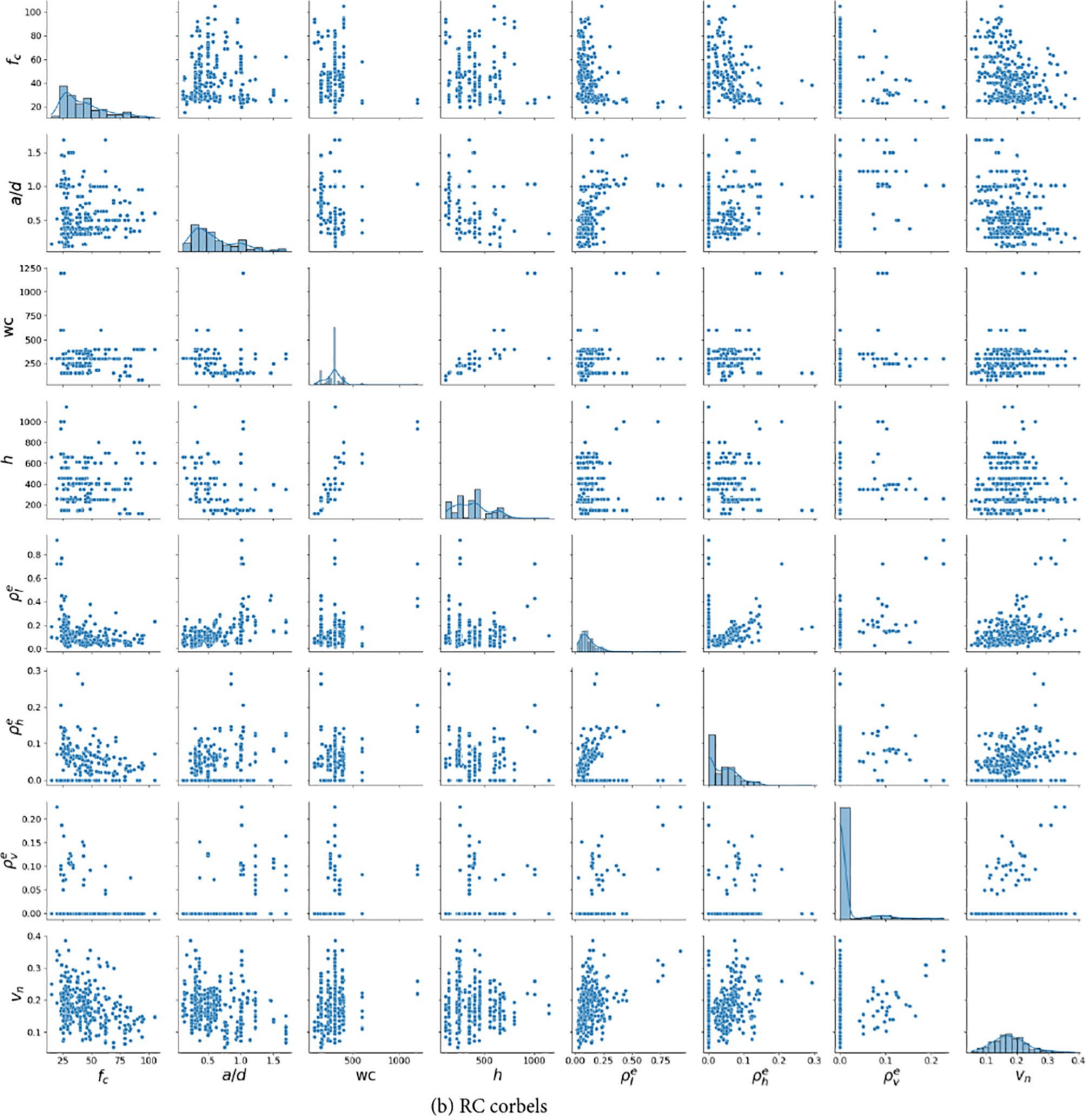
Distribution of the databases and the relationships between different parameters.

In this study, the min-max scaling technique is applied to normalize the data and minimize the negative impacts of multidimensionality. The normalized datasets are then randomly split into two sets, with 80% used for training the model and 20% reserved for testing. The statistical distributions of these databases and relationships between different parameters are presented in Fig. 5.

The performance of proposed SR-STM model is assessed by comparing it with the ML-based CatBoost model and several mechanical models, including those developed by Hwang and Lee^18^, Russo et al^11,17^. and Chetchotisak et al^19^.. The design equations for these three mechanical models are listed in Table 4.

**Figure 6:**
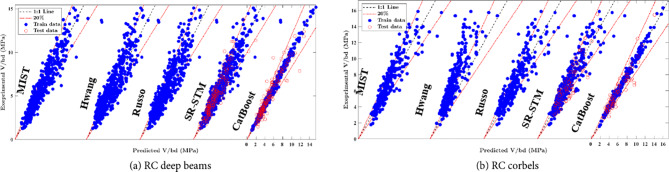
Comparison between proposed equations and previous models.

Figure 6 shows scatter plots that compare experimental and predicted results for the SR-STM, CatBoost, and mechanical models. For both the SR-STM and CatBoost models, most data points are closely aligned along the diagonal line, indicating a high level of accuracy and reliability in the predictions made by these models. This close correlation between the predicted and actual results highlights the strong performance of the SR-STM and CatBoost models in accurately estimating the shear strength of RCDBs and RCCs. Detailed evaluation metrics used to assess the performance of these models are presented in Tables 5 and 6. These metrics include the mean (*µ*), coefficient of variance (CoV), coefficient of determination (*R*^*2*^), root mean squared error (RMSE), mean absolute percentage error (MAPE), and a20-index, as defined below:

**Table 5:** Comparison of the developed ML models for RC deep beams.

Metrics	Training data		Testing data		All data				
	CatBoost	SR-STM	CatBoost	SR-STM	CatBoost	SR-STM	Hwang^18^	Russo^11^	Chetchotisak (MIST)^19^
Mean $\:\mu\:$	0.999	0.993	1.002	1.024	0.999	0.999	1.056	0.979	0.989
%CoV	4.56	14.11	14.80	15.86	7.79	14.55	18.75	18.48	15.24
R^2^	0.994	0.919	0.908	0.879	0.979	0.913	0.827	0.870	0.906
MAPE	3.295	10.89	10.67	13.84	4.770	11.48	16.51	14.58	11.95
RMSE(kN)	0.213	0.913	0.758	0.957	0.389	0.922	1.300	1.128	0.956
a20-index	0.998	0.86	0.833	0.772	0.965	0.842	0.684	0.731	0.828

**Table 6:** Comparison of the developed ML models for RC corbels.

Metrics	Training data		Testing data		All data				
	CatBoost	SR-STM	CatBoost	SR-STM	CatBoost	SR-STM	Hwang^18^	Russo^17^	Chetchotisak (MIST)^19^
Mean $\:\mu\:$	0.999	1.013	1.000	0.967	0.999	1.004	1.322	1.010	1.016
%CoV	4.71	14.19	13.01	17.36	7.19	14.95	18.68	18.45	16.18
R^2^	0.989	0.876	0.873	0.780	0.974	0.862	0.448	0.799	0.807
MAPE	3.465	11.776	10.00	14.16	4.769	12.25	34.03	14.69	13.18
RMSE(kN)	0.303	1.185	0.843	1.338	0.464	1.217	2.43	1.468	1.437
a20-index	0.997	0.815	0.865	0.676	0.97	0.787	0.329	0.714	0.757

**Table:** 

Algorithm 1. Objective function.	
**function** objective_function(*tree*,* data*): Initialize penalty term *P* = 0 **if** tree.degree ≠ 2 then *P* + = 1000 **else** *TL* = *tree.l* #left tree **if ***TL*.degree ≠ 2 then *P* + = 1000 **else** *TLL = TL.l* # left subtree of left tree *P* + = 100 * NOVs(*TLL* ≠ $\:f\left({f}_{c}^{{\prime\:}},{k}_{s}\right)$, *TLL* < 0)^**^ $\:{v}_{s}$= eval_tree(*TLL*,* data.x*) *TLR = TL.r* # right subtree of left tree *P* + = 100 * NOVs(*TLR* ≠ $\:f\left({\rho\:}_{l}^{e}\right),$*TLR* < 0) $\:{v}_{t}$= eval_tree(*TLR*, *data.x*) $\:{v}_{c}$= $\:\frac{1.0}{\frac{1.0}{{v}_{s}*data.x\left[{sin}\theta\:\right]}\:+\:\frac{1.0}{{v}_{t}*data.x\left[{tan}\theta\:\right]}}$ # **concrete contribution** **end if** *TR = tree.l* #left tree **if ***TR.degree* ≠ 2 then *P* + = 1000 **else** *TRL = TR.l* # left subtree of right tree *P* + = 100 * NOVs(*TRL* ≠ $\:f\left({\rho\:}_{h}^{e}\right)$, *TRL* < 0) $\:{v}_{h}$= eval_tree(*TRL*,* data.x*) *TRR = TR.r* # right subtree of right tree *P* + = 100 * NOVs(*TRR* ≠ *f*($\:{\rho\:}_{v}^{e}$), *TRR* < 0) $\:{v}_{v}$= eval_tree(*TRR*,* data.x*) $\:{v}_{hv}={v}_{h}$*data.x$\:\left[{tan}\theta\:\right]+{v}_{v}$ # **web reinforcement contribution** **end if** *r* = *dataset.y*/($\:{v}_{c}+{v}_{hv}$) # True-to-pred ratio return $\left(\frac{sum\left(abs.\left(r-1\right)\right)}{length\left(r\right)}+P\right)$ # **Return MAPE + penalty term** **end function**	

**: The expression NOV(TLL ≠ $\:f\left({f}_{c}^{{\prime\:}},{k}_{s}\right)$ && TLL < 0) quantifies the number of violations where the TLL subtree is not a function of the features $\:{f}_{c}^{{\prime\:}}$ and $\:{k}_{s}$, or where it includes other features, or when its value is negative.

22 $$\begin{aligned}{R}^{2}=1-\frac{\sum_{i=1}^{n}{\left({\widehat{y}}_{i}-{y}_{i}\right)}^{2}}{\sum_{i=1}^{n}{\left({y}_{i}-\stackrel{-}{y}\right)}^{2}},\:\:\mu\:=\frac{1}{n}\sum_{i=1}^{n}\frac{{\widehat{y}}_{i}}{{y}_{i}},\:\:\:\:\:MAPE=\frac{100\%}{n}\sum_{i=1}^{n}\left|\frac{{\widehat{y}}_{i}}{{y}_{i}}-1\right|,\:\:RMSE=\sqrt{\frac{1}{n}\sum_{i=1}^{n}{\left({\widehat{y}}_{i}-{y}_{i}\right)}^{2}}\end{aligned}$$

In this context, $\:{y}_{i}$ refers to the predicted value for the *i*-th specimen, $\:{\widehat{y}}_{i}$ is the corresponding actual output value, $\:\stackrel{-}{y}$​ is the average of the actual observations, and n indicates the total number of samples in the database. The a20-index^33^ assesses the percentage of specimens where the ratio $\:{\widehat{y}}_{i}/{y}_{i}$ falls within the range of 0.80 to 1.20.

As demonstrated in Tables 5 and 6, both SR-STM and CATB models exhibit high accuracy, with mean *µ*, *R*^*2*^, and a20-index values approaching 1.0, along with low CoV, MAPE, and RMSE. Specifically, the CATBoost model achieves MAPE values of approximately 3.3% and 10.7% for RC deep beams and 3.5% and 10% for RC corbels in the training and testing sets, respectively, which are the lowest among the compared models. The SR-STM model, while slightly less accurate with MAPE values of 10.84% and 11.76% for RC deep beams and 13.84% and 14.16% for RC corbels, still provides a strong performance. Furthermore, the introduced SR-STM model yields *µ* values of 0.999 and 1.004, *R*^*2*^ values of 0.913 and 0.862, and CoV values of 14.55% and 14.95% for RC deep beams and RC corbels, respectively. Although the SR-derived formulas show slightly lower accuracy compared to the CATBoost (CATB) model, they are more accessible and easier to interpret, which significantly enhances their practical utility in engineering applications. While the CATBoost model demonstrates superior prediction accuracy, its black-box nature restricts its practical application in engineering design. In contrast, the SR-STM model provides a more interpretable approach that bridges the gap between theoretical insight and practical use.

## Comparisons with closed-form models

In this section, the proposed equations are compared with three existing closed-form models listed in Table 4, including the models by Hwang and Lee^18^, Russo et al^11,17^., and Chetchotisak et al. (MIST)^19^ models. Tables 5 and 6 provide statistical information on the predictive capabilities of these models for RC deep beams (RCDBs) and RC corbels (RCCs), respectively. The proposed equations yield values of *µ*and CoV of 0.999 and 14.55% for RCDBs and 1.004 and 14.95% for RCCs. In contrast, the existing closed-form models display CoV greater than 18%, except for Chetchotisak et al^19^., which shows CoV values of 15.24% and 16.18% for RCDBs and RCCs, respectively. This indicates that the proposed equations exhibit better predictive stability and robustness compared to the existing models.

The models introduced by Russo^11,17^and Chetchotisak^19^show mean values with slight deviations from 1.0. In contrast, the Hwang and Lee model^18^ shows the highest mean deviation for RCCs, with a mean value of 1.322. Figure 6introduces scatter plots illustrating the relationship between experimental and predicted results across the entire database using the proposed equations and the three closed-form models. The SR-STM proposed in this study displays prediction-to-test ratios concentrated around unity, achieving the highest accuracy among the previous models. Furthermore, the CATBoost model demonstrates superior performance, with CoV values of 7.79% for RCDBs and 7.19% for RCCs, highlighting the effectiveness of using ML techniques for shear strength prediction. On the other hand, the prediction results of closed-form models reveal deficiencies in their prediction mechanisms. In particular, the Hwang expression^18^ for RCCs shows the most significant deviation between experimental and predicted values, with a distribution skewed over the diagonal line, indicating a tendency toward conservative predictions. The improvement of SR-STM reflects not only higher prediction accuracy compared to closed-form models but also in providing more specific physical significance and mathematical equations compared to purely data-driven models e.g., the CATB model.

**Figure 7:**
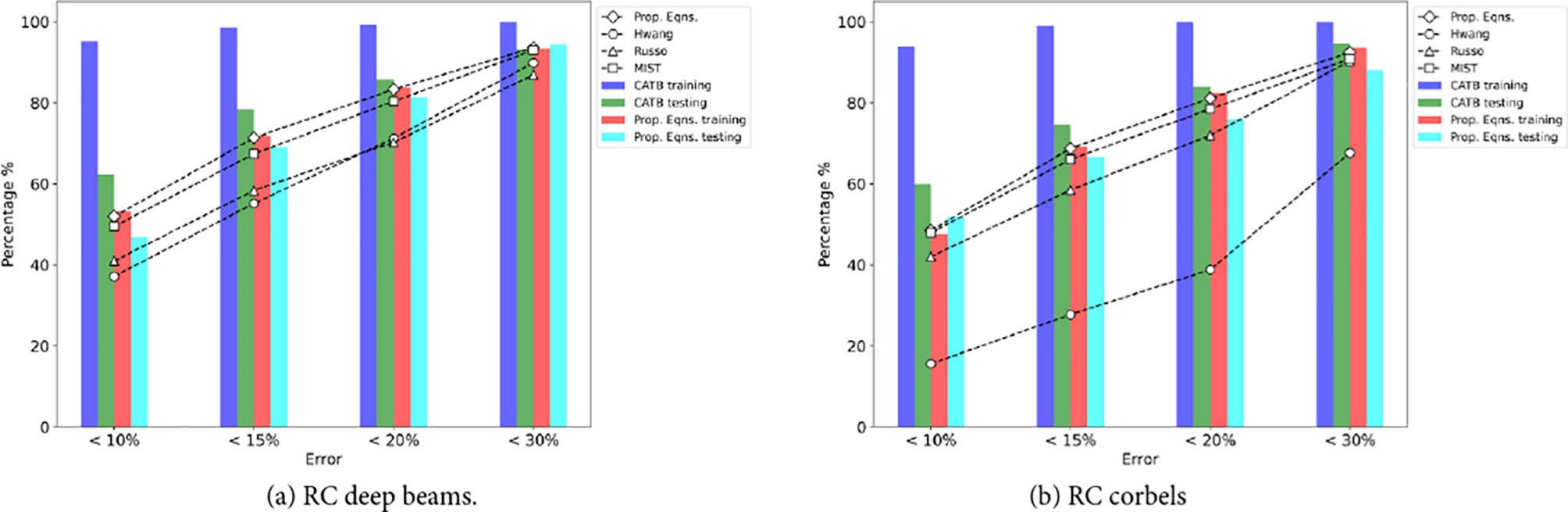
Prediction errors of previous models and established ML models.

Figure 7 displays the prediction errors of both existing closed-form expressions and the developed ML models. In Fig. 7(a), the CATB and ST-STM models exhibit high precision, with over 83% of testing samples within a 20% error range. In contrast, the Hwang and Russo formulas have almost 70% of samples within the same range. The MIST model also performs well, capturing 80% of samples within the 20% error range. In Fig. 7(b), the ST-STM and MIST formulas for RCCs show similar performance, with a slight advantage for the ST-STM. While the results of the proposed equations and the MIST formula are comparable, the proposed equations are more straightforward to implement. In addition, the ST-STM significantly outperforms the Hwang model by having nearly three times the number of test samples within the same error ranges for RC corbels. Moreover, all performance metrics for the introduced SR-STM, as detailed in Tables 5 and 6, exceed those of the previously introduced mechanical models. These results highlight the superior performance of employing ML techniques, such as the CATB and ST-STM models, in predicting the shear strength of RC deep beams and RC corbels.

## Feature importance analysis

**Figure 8:**
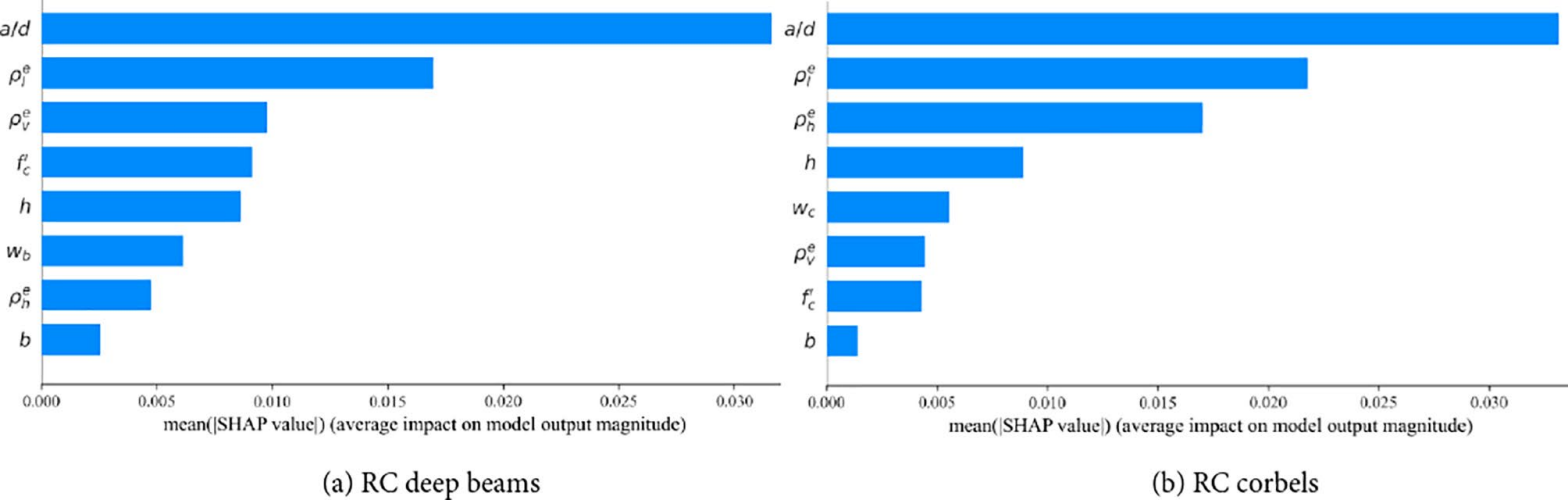
Summary plot for inputs influencing shear strength index *V*_*n*_*/b*_*w*_*d f*_*c*_*’* .

Assessing the impact of input parameters on the shear strength is crucial for designing RCDBs and RCCs. This study utilizes the Shapley Additive Explanation (SHAP) method to identify and highlight the most significant parameters influencing the shear strength index, *V*_*n*_*/b*_*w*_*d f*_*c*_*’*^34^. As depicted in Fig. 8, the summary plot reveals the effect of each feature on model predictions and ranks the features according to their relative importance on the axial strength index. The feature with the highest absolute SHAP value is considered the most significant. The span-to-depth ratio (*a/d*), and effective longitudinal reinforcement ratio ($\:{\rho\:}_{l}^{e}$) emerge as the most significant parameters for both RCDBs and RCCs. Additionally, the analysis shows that the effective vertical and horizontal web reinforcement ratios ($\:{\rho\:}_{v}^{e}$, $\:{\rho\:}_{h}^{e}$) rank as the third most important feature for RCDB and RCC databases, respectively. The significance of the remaining features is ranked in descending order.

**Figure 9:**
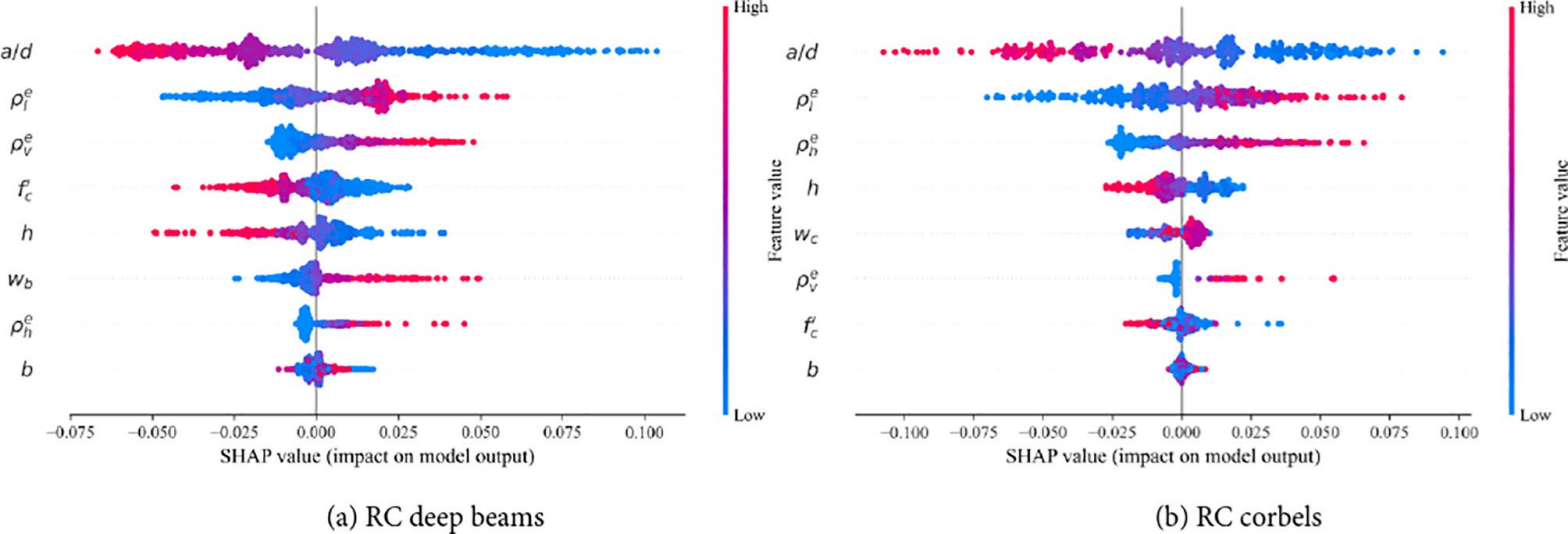
Features importance for inputs influencing shear strength index *V*_*n*_*/b*_*w*_*d f*_*c*_*’*.

Figure 9 illustrates the SHAP feature importance for each input variable in the RCDBs and RCCs databases. A feature importance value greater than zero indicates a positive correlation with the strength index, whereas a value less than zero suggests a negative impact. The length and color of the bars in Fig. 9 represent the significance and direction (positive or negative) of each feature, respectively. It is evident that, except for the *a/d* ratio, concrete strength *f*_*c*_*’*, and height *h*, the remaining input features have a positive and mixed influence on the shear strength index. Increasing effective reinforcement ratios ($\:{\rho\:}_{l}^{e}$, $\:{\rho\:}_{v}^{e}$, $\:{\rho\:}_{h}^{e}$), top plate width for RCDBs *w*_*b*_, and column width for RCCs *w*_*c*_, will improve the shear strength index. Conversely, the *a/d* ratio, concrete strength *f*_*c*_*’*, and height *h* negatively impact the shear strength index. The negative impact of the *a/d*ratio aligns with experimental findings by Kani^32^, which show that beams exhibit higher shear resistance at lower *a/d* values. Moreover, increasing the beam height *h*, reduces the shear resistance, as deeper beams weaken shear transfer strength through aggregate interlock along critical shear cracks, resulting in higher energy release and further reduction in shear resistance^35^.

The equation extracted from the SR-STM model aligns well with the results obtained from the SHAP analysis, reinforcing the reliability and interpretability of the SR-STM model. As shown in Eqs. (18), (20), and (21) of the SR-STM model, the concrete strength *f*_*c*_*’* has a negative impact on the shear strength index *V*_*n*_*/b*_*w*_*d f*_*c*_*’*, consistent with the SHAP analysis results. Additionally, the SR-STM model demonstrates that increasing the effective reinforcement ratios ($\:{\rho\:}_{l}^{e}$, $\:{\rho\:}_{v}^{e}$, $\:{\rho\:}_{h}^{e}$), top plate width for RCDBs *w*_*b*_, column width for RCCs *w*_*c*_, and angle *θ* (which is proportional to the inverse of the span-to-depth ratio *a/d*) enhances the shear strength index. This positive effect is also reflected in the SHAP analysis, where these parameters increase the predicted shear strength. Therefore, the proposed equation’s behavior is consistent with the SHAP analysis, highlighting the model’s ability to accurately capture the relationships between the input features and the shear strength of RCDBs and RCCs.

### Limitations and future work

This section addresses the limitations of the SR-STM model and highlights potential areas for future research. While the SR-STM model shows strong predictive capabilities, its applicability is inherently limited by the scope of the data used for its development. The model is tailored to specific geometries and material properties within the dataset, as detailed in Table 2. For instance, the model has been developed based on beams with *a/d* ratios ranging from 0.27 to 2.5 for RCDBs and 0.11 to 1.69 for RCCs and on concrete strength between 11.3 MPa and 120.1 MPa for RCDBs and 15 MPa to 105 MPa for RCCs. The reinforcement ratios and yield strengths also cover specific ranges, which may not cover all real-world scenarios. Future work could focus on expanding the dataset to include a broader range of geometries and material properties. As a result, applying the SR-STM model for beams or corbels with geometries or material properties outside these ranges may lead to inaccurate predictions, as it would involve extrapolation beyond the model’s trained data.

The evolving field of energy-harvesting concrete marks a significant step toward smart, sustainable infrastructure. This technology integrates materials that allow concrete structures to harvest and store energy, creating multifunctional, self-sustaining elements^36–39^. Machine learning, particularly symbolic regression, can optimize the performance of these materials, enhancing the efficiency of energy-harvesting concrete. By incorporating energy-harvesting considerations into the design, we can develop concrete structures that are both structurally sound and energy-efficient. This approach could lead to self-powered roadways, buildings, and bridges, advancing sustainable construction practices.

## Conclusions

In conclusion, this study compiled a comprehensive database of 810 experimental tests for the shear strength of RC deep beams (RCDBs) and 371 RC corbels (RCCs) tests from various research papers. It employed symbolic regression (SR) techniques to refine and calibrate the Strut-and-Tie Model (SR-STM). From the evaluation results, the following conclusions can be drawn:

- The integration of symbolic regression with the Strut-and-Tie Model successfully enhances prediction accuracy while maintaining the interpretability and consistency of the models with established mechanical principles.
- The SR-STM model yields µ values of 0.999 and 1.004, R² values of 0.913 and 0.862, and CoV values of 14.55% and 14.95% for RC deep beams and RC corbels, respectively, indicating high predictive stability and robustness.
- Compared to existing closed-form models by Hwang and Lee^18^, Russo et al^11,17^., and Chetchotisak et al. (MIST)^19^, the SR-STM model shows better predictive performance, with improved CoV values and concentrated prediction-to-test ratios around unity.
- While the CATBoost model demonstrates superior performance with CoV values of 7.79% for RCDBs and 7.19% for RCCs, its black-box nature limits practical application in engineering design, highlighting the need for more interpretable models like SR-STM.
- The SR-STM model significantly outperforms the Hwang model by having nearly three times the number of test samples within the 20% error range for RC corbels, and it surpasses all performance metrics compared to previously introduced mechanical models.
- The alignment between the SR-STM model’s equations and the SHAP analysis confirms the model’s effectiveness in accurately capturing the key factors influencing the shear strength of RCDBs and RCCs.

The SR-STM not only achieves significant improvements in shear strength prediction but also effectively combines the advantages of white-box and black-box models. Compared to purely data-driven approaches, the SR-STM, with its explicit mathematical equations, is more accessible and reliable for engineers to utilize in practical applications. While the SR-STM model demonstrates strong predictive capabilities, its effectiveness is limited by the dataset’s scope, necessitating future research to broaden the range of geometries and material properties to enhance model applicability and accuracy. In summary, integrating the ML-based approach presents a promising method for accurately predicting the shear strength of RC deep elements, providing valuable insights for engineering applications.

## Electronic supplementary material

Below is the link to the electronic supplementary material.

Supplementary Material 1

## Data Availability

All data generated or analyzed during this study are included in this published article and available in a public repository: https://github.com/kmegahed/SR-STM.
